# Reporting of academic degrees in high-impact medical journals

**DOI:** 10.12688/f1000research.21096.3

**Published:** 2020-02-20

**Authors:** Nikola Stankovic, Jerry P. Nolan, Lars W. Andersen

**Affiliations:** 1 Research Center for Emergency Medicine, Department of Clinical Medicine, Aarhus University and Aarhus University Hospital, Aarhus, Denmark; 2 Royal United Hospital, Bath, UK

**Keywords:** Academic, degree, impact factor, journals, publication, high-impact

## Abstract

Academic degrees following author names are often included in medical research papers. However, it remains unclear how many journals choose to include academic degrees and whether this is more common in certain types of journals. We examined the 100 highest impact medical journals and found that only 24 medical journals reported academic degrees. Moreover, this was substantially more common in journals based in North America compared with Europe. Further research is required to explore the implications of listing academic degrees on the readers’ attitude towards research quality.

## Introduction

During submission of research papers to medical journals, authors are often asked to include academic degrees and/or affiliations. However, journals differ in how they present this information to readers. While some journals include academic degrees following author names, others choose not to list this information on the title page. To our knowledge, no evidence is available on how many journals choose to include academic degrees and whether this is more common in certain types of journals. Among the most influential medical journals, we examined journal factors associated with the inclusion of academic degrees on the title page.

## Methods

We identified the 100 highest impact medical journals based on impact factor reported in the Journal Citation Reports ^1^ published in 2018. These journals constituted 4% of the total number of medical journals identified in Journal Citation Reports. Characteristics of each journal in regard to specialty, impact factor, primary journal focus, continent, and open access policy were obtained. Journals were categorized as “general” or “specific” based on subject area designation in Scopus ^2^, i.e. whether content was primarily related to one specialty (e.g. cardiology) or multiple specialties. Data were collected on the presence of academic degrees following author names in the title page by assessment of multiple original research articles from each journal. The presence of academic degrees was defined as the inclusion of academic degree abbreviations following author names (e.g. MD, PhD, RN, MPH etc.). Similarly, we collected data on fellowship designations and job titles following author names. Multiple articles published throughout July 2018 and August 2019 were assessed for each journal. No discrepancy of reporting academic degrees throughout the time period was identified. If there was any discrepancy between the print and the online version, the print version was used. Open access was identified based on open access designation in Scopus. Scopus registers open access as follows: “ *In Scopus, journals are registered as being OA journals only if they are registered as Gold OA or Subsidized OA at one or both of the following sources: Directory of Open Access Journals (DOAJ) and the Directory of Open Access Scholarly Resources (ROAD)*” ^3^.

Descriptive statistics were used to characterize the journals. Categorical data were compared with the Fisher’s Exact Test and continuous data were compared with the Wilcoxon Rank-Sum Test. The association between journal characteristics and the reporting of academic degrees were estimated using multivariable logistic regression.

Statistical analyses were performed in SAS (version 9.4). A two-tailed p < 0.05 was considered significant.

## Results

Of the 100 highest impact medical journals, 24 journals reported academic degrees on the title page ( Table 1). We found that 52% of journals were published in Europe and 48% were published in North America. Only 8% of European journals reported academic degrees while 42% of North American journals reported academic degrees. The median impact factor of journals reporting and not reporting academic degrees was 12 (IQR 10 – 19) and 15 (IQR 11 – 20), respectfully. Moreover, six journals reported fellowship designations and one journal reported job titles (online only), all of which included academic degrees.

**Table 1.** The presence of academic degree(s) according to journal factors in the top 100 high-impact medical journals.

		Presence of academic degree		Multivariable model	
		Yes (n = 24)	No (n = 76)	OR	95%CI
Primary journal focus	Clinical	17 (71%)	26 (34%)	Ref.	-
	Basic science and/or experimental	0 (0%)	21 (28%)	NA ^a^	NA ^a^
	Combined	7 (29%)	28 (37%)	0.19	0.05 - 0.80
Specialty	General	4 (17%)	9 (12%)	Ref.	-
	Specific	20 (83%)	67 (88%)	1.77	0.27 - 11.54
Open access	Yes	1 (4%)	6 (8%)	Ref.	-
	No	23 (96%)	70 (92%)	4.94	0.41 - 59.36
Continent	Europe	4 (17%)	48 (63%)	Ref.	-
	North America	20 (83%)	28 (37%)	19.95	4.71 - 84.44
Journal impact	Impact factor ^b^	12 (10 – 19)	15 (11 – 20)	1.00	1.00 - 1.00

^a^Not able to be estimated as no journal in this category reported academic degrees

^b^Median with quartiles Multivariable analysis showed that North America and a clinical journal focus was associated with increased odds of reporting academic degrees ( Table 1). No association was found for the other journal characteristics.

## Discussion

Among the 100 highest impact medical journals, only 24 journals reported academic degrees following author names on the title page. Reporting of academic degrees was substantially more common in journals based in North America compared with Europe.

Listing author academic degrees is an editorial policy decision but there is little guidance from the International Committee of Medical Journals Editors (ICMJE) or the American Medical Association (AMA) Manual of Style. Specifically, the ICMJE states “ *Each author's highest academic degrees should be listed, although some journals do not publish these* ^4^.” and the AMA Manual of Style writes “ *Journals should establish their own policies on the inclusion of authors' degrees.*” ^5^. Neither provides a rationale for providing academic degrees and it remains unclear why some journals do and others do not. The marked difference between journals published in North America compared with Europe cannot be explained by the current study but may be a reflection of cultural differences in attitude towards degrees and titles. To our knowledge, no studies exist on the impact of reporting academic degrees in medical journals. Further research is needed to explore 1) trends in reporting academic degrees, 2) the implications of listing academic degrees on the readers’ attitude towards research quality, and 3) trends in academic degree characteristics considering that specific journals have seen an increase in authors with bachelor’s and multiple academic degrees ^6– 8^.

Limitations of the current study include that we only evaluated high-impact medical journals. Furthermore, we only assessed some journal characteristics. We evaluated only recent issues of these journals and are therefore unable to comment on trends in the use of author academic degrees.

In conclusion, we found that academic degrees are reported in about one fourth of medical journals and that this practice is more common in North America.

## Data availability

### Underlying data

Harvard Dataverse: Replication Data for: Reporting of academic degrees in high-impact medical journals, https://doi.org/10.7910/DVN/KTWS6C ^9^

This project contains the following underlying data:

- CSV file with the titles of the medical journals investigated

Data are available under the terms of the Creative Commons Zero "No rights reserved" data waiver (CC0 1.0 Public domain dedication).

The Journal Citation Report from Clarivate Analytics can only be accessed through an individual or institutional account.

## Notes

^1^ https://jcr.clarivate.com/JCRLandingPageAction.action Accessed September 7 ^th^ 2019

^2^  Accessed Dec. 5th 2019

^3^  Assessed December 5 ^th^ 2019

^4^ http://www.icmje.org/recommendations/browse/manuscript-preparation/preparing-for-submission.html#a Accessed September 7 ^th^ 2019

^5^ https://www.amamanualofstyle.com/view/10.1093/jama/9780195176339.001.0001/med-9780195176339-div2-10 Accessed September 7 ^th^ 2019

^6^Haws BE, Khechen B, Movassaghi K, *et al.* Authorship Trends in Spine Publications From 2000 to 2015. Spine (Phila Pa 1976). 2018;43(17):1225–1230

^7^Schrock JB, Kraeutler MJ, McCarty EC. Trends in Authorship Characteristics in The American Journal of Sports Medicine, 1994 to 2014. Am J Sports Med. 2016;44(7):1857–1860

^8^Gu A, Almeida N, Cohen JS, Peck KM, Merrell GA. Progression of Authorship of Scientific Articles in The Journal of Hand Surgery, 1985–2015. J Hand Surg Am. 2017;42(4):291 e291–291 e296.

^9^Stankovic, Nikola, 2019, "Replication Data for: Reporting of academic degrees in high-impact medical journals", https://doi.org/10.7910/DVN/KTWS6C, Harvard Dataverse, V1
