# Supplementary material for: Reporting of academic degrees in high-impact medical journals
Source: F1000Res. 2020 Feb 20;8:1852. Originally published 2019 Nov 5. [Version 3] doi: 10.12688/f1000research.21096.3 (PMC7014575; doi:10.12688/f1000research.21096.3)
Supplement: Peer review history

# Peer review: Reviewer response for version 3

**DOI:** 10.5256/f1000research.24841.r60328

**Published:** 2020-02-21

**Authors:** Ana Marusic

**Affiliations:** 1 Department of Research in Biomedicine and Health, School of Medicine, University of Split, Split, Croatia

The authors have addressed my comments and suggestions.

I confirm that I have read this submission and believe that I have an appropriate level of expertise to confirm that it is of an acceptable scientific standard.

# Peer review: Reviewer response for version 2

**DOI:** 10.5256/f1000research.23852.r58696

**Published:** 2020-02-11

**Authors:** Christopher C. Winchester

**Affiliations:** 1 Oxford PharmaGenesis Ltd, Oxford, UK

Thank you for the opportunity to review the work of Stankovic and colleagues on the prevalence of reporting of academic degrees on the title pages of 100 leading medical journals selected based on journal impact factor. The resulting brief communication is short, clear and to the point.

 My main question is why the authors undertook the study, and what they make of the findings? Do they believe that authors should receive public credit for the hard work involved in their studies? Do they believe that readers should take into account authors’ academic degrees when interpret research findings? Or do they find collecting and submitting information on academic degrees burdensome and pointless (particularly if it was never actually published with the article)? Or think that research should be judged on its merits and not on the qualifications of its authors? The latter point is particularly pertinent given the increasing discussion of including patients as authors on articles in medical journals. ^1^^,^^2^^,^^3^ Readers of this brief communication will be interested to hear the authors’ take on their findings.

 I have some minor comments below:

- What was the impact factor of the top 100 journals? It would be helpful to know if the impact factor cut-off was 1 or 10. Or, to look at it a different way, how many medical journals are listed in total, and what % of those journals was included? This could be mentioned in the Methods or when discussing generalizability. While considering generalizability, the fact that the selected journals included both general and specialist journals, from both the US and Europe, and published under both open access and subscription models, is a clear strength of the study.
- Journals were characterized as ‘general’ or ‘specific’ – the authors should consider the alternative term ‘specialist’.
- The information the authors collected on fellowship designations and job titles is of interest. As the authors have collected it, perhaps it could include these in the main article on a descriptive basis, as a single short sentence in the Methods and Results.
- The following sentence needs splitting in two:
  “To our knowledge, no studies exist on the impact of reporting academic degrees in medical journals, why further research is needed to explore the implications of listing academic degrees on the readers’ attitude towards research quality.”
  For example:
  “To our knowledge, no studies exist on the impact of reporting academic degrees in medical journals. Further research is needed to explore the implications of listing academic degrees on readers’ perceptions of research quality.”
- Another idea for further research would be to study trends in the prevalence of reporting of academic degrees in medical journals. Furthermore, it would be interesting to look at how the degrees of authors (in journals that report them) are changing over time. Perhaps you could include these ideas among your avenues for further research, and cite the references suggested by Marusic, which suggest that the number of authors with a Bachelor’s degree as their highest degree is increasing.

Thanks again for the chance to review this interesting and worthwhile manuscript.

I confirm that I have read this submission and believe that I have an appropriate level of expertise to confirm that it is of an acceptable scientific standard.

## References

[1] : One small step…. *Res Involv Engagem*.2015;1: 10.1186/s40900-015-0005-8 1 10.1186/s40900-015-0005-8 29062499PMC5611604

[2] : Frequency of reporting on patient and public involvement (PPI) in research studies published in a general medical journal: a descriptive study. *BMJ Open*.2018;8(3) : 10.1136/bmjopen-2017-020452 10.1136/bmjopen-2017-020452 PMC587563729572398

[3] : Patient Involvement in Publications of Industry-Sponsored Research: Cross-Sectional Survey. *Curr Med Res Opin*.2018;34(Suppl 1)(24) : 10.6084/m9.figshare.6493103.v1 10.6084/m9.figshare.6493103.v1

# Response

**Published:** 2020-02-18

**Authors:** Lars Andersen

**Affiliations:** Aarhus University Hospital, Denmark

Thank you for raising some important questions along with adding insights into patient and public involvement in research through your suggested articles. Overall, our manuscript adds insights to the reporting of academic degrees in high-impact medical journals. However, it does not add insights to additional reasons for including academic degrees or authors’ perception of the submission process. We understand why the reviewer and readers may be interested in our take on the findings of the study. Despite this, we believe that it is necessary to keep this manuscript strictly descriptive, since any opinion on the raised questions may be considered to be of speculative nature. However, we believe that it stresses the need for additional research to address these questions scientifically. 

In the results section, we previously included that the median impact factor was 12 in journals reporting academic degrees and 15 in journal not reporting academic degrees. Upon review of your comments, we decided to include following sentence: “These journals constituted 4% of the total number of medical journals identified in Journal Citation Reports.”

We agree that the term ‘specialist’ could be used when characterizing journals. However, we chose to characterize journals as ‘specific’ as they relate to a certain subject area. We believe that the term ‘specialist’ may cause equivocality to some readers, since journals who report on more general subject areas also provide specialist knowledge and research. Thus, we chose to keep the term ‘specific’. 

We included a few concise statements regarding fellowship designations and job titles in the Methods and Results sections as follows:

Methods: “Similarly, we collected data on fellowship designations and job titles following author names.”

Results: “Moreover, six journals reported fellowship designations and one journal reported job titles (online only), all of which included academic degrees.”

We agree that further research is needed to address trends in the prevalence of reporting academic degrees and the characteristics of the academic degrees. Thus, we included following in the Discussion section: “Further research is needed to explore 1) trends in reporting academic degrees, 2) the implications of listing academic degrees on the readers’ attitude towards research quality, and 3) trends in academic degree characteristics considering that specific journals have seen an increase in authors with bachelor’s and multiple academic degrees.”

# Peer review: Reviewer response for version 2

**DOI:** 10.5256/f1000research.23852.r57744

**Published:** 2019-12-16

**Authors:** Ana Marusic

**Affiliations:** 1 Department of Research in Biomedicine and Health, School of Medicine, University of Split, Split, Croatia

The authors addressed most of my comments and concerns, but I am still not sure that it is appropriate not to present references that studied academic degrees and authorship. While there is no overview of the practices in different journals, there is certainly research in individual journals. In biomedicine see here ^1^^-^^4^.

 These studies justify the need for a comparison study and they also show that there can be valuable information if academic degrees are published in a journal article. I suggest to address these points.

I confirm that I have read this submission and believe that I have an appropriate level of expertise to confirm that it is of an acceptable scientific standard, however I have significant reservations, as outlined above.

## References

[1] : Authorship Trends in Spine Publications From 2000 to 2015. *SPINE*.2018;43(17) : 10.1097/BRS.0000000000002585 1225-1230 10.1097/BRS.0000000000002585 29419716

[2] : Trends in Authorship Characteristics in The American Journal of Sports Medicine, 1994 to 2014. *Am J Sports Med*.2016;44(7) : 10.1177/0363546516639955 1857-60 10.1177/0363546516639955 27159311

[3] : Progression of Authorship of Scientific Articles in The Journal of Hand Surgery, 1985-2015. *J Hand Surg Am*.2017;42(4) : 10.1016/j.jhsa.2017.01.005 291.e1-291.e6 10.1016/j.jhsa.2017.01.005 28185698

[4] : Authorship Trends of Emergency Medicine Publications over the Last Two Decades. *West J Emerg Med*.2016;17(3) : 10.5811/westjem.2016.2.29779 367-71 10.5811/westjem.2016.2.29779 27330673PMC4899072

# Response

**Published:** 2019-12-17

**Authors:** Lars Andersen

**Affiliations:** Aarhus University Hospital, Denmark

Overall, the suggested articles deals with trends in authorship and academic degree characteristics in specific medical journals. Specifically, studies 1-3 found an increase in authors per article and changes in the distribution of academic degrees among authors within their respective journals. Study 4 examined trends in publication from allopathic and osteopathic physicians, respectively. None of the studies directly dealt with the question we studied. 

Thus - after review of the suggested articles – we have decided not to incorporate the articles, since they are not directly relevant to the topic.

# Peer review: Reviewer response for version 2

**DOI:** 10.5256/f1000research.23852.r57745

**Published:** 2019-12-13

**Authors:** Saif Aldeen AlRyalat

**Affiliations:** 1 Department of Ophthalmology, University of Jordan Hospital, The University of Jordan, Amman, Jordan

I would like to applaud the authors' efforts in thoroughly reviewing their article. Again, this study raised a good insight into the importance of considering reporting academic degrees of authors as part of their affiliation in medical journals. I also agree that future studies are needed to assess the impact of reporting academic degrees of authors on the reader's behavior toward the article quality as well as on authors' satisfaction regarding the content of their affiliations.

I confirm that I have read this submission and believe that I have an appropriate level of expertise to confirm that it is of an acceptable scientific standard.

# Peer review: Reviewer response for version 1

**DOI:** 10.5256/f1000research.23217.r56491

**Published:** 2019-11-18

**Authors:** Ana Marusic

**Affiliations:** 1 Department of Research in Biomedicine and Health, School of Medicine, University of Split, Split, Croatia

This article addresses an interesting and intriguing question in publishing - why author's academic degrees are published in journals. Editorial guidelines mention this, but do not provide actual guidance.

 This research showed that journals from North America and from clinical disciplines are more likely to publish the degrees, which is an interesting finding, at least in high-impact journals.

 The manuscript is written clearly, as a brief report, and follows a logical order, with appropriate statistical analysis.

 I have two major issues:

1. There is no literature overview on this problem (or a statement that there is no evidence on this issue in literature).
2. The description of the selection of articles in the set of 100 journals is vague. It is not clear what was the method for sampling "approximately ten articles ..." in the two study years. This needs to be clarified. 

I confirm that I have read this submission and believe that I have an appropriate level of expertise to state that I do not consider it to be of an acceptable scientific standard, for reasons outlined above.

# Response

**Published:** 2019-12-06

**Authors:** Lars Andersen

**Affiliations:** Aarhus University Hospital, Denmark

**Comment 1:**

- “There is no literature overview on this problem (or a statement that there is no evidence on this issue in literature).”

 

**Response 1: **

- Thank you for raising an important point in the manuscript. To our knowledge – following literature review – there are no studies evaluating the impact of reporting academic degrees following author names in medical journals. We agree that a statement addressing that no evidence on this issue is available, will add valuable content to the manuscript. It should be noted, however, that the current study does not evaluate *why* journals report academic degrees or whether it has an impact on the readers. In this study, we evaluated whether high-impact medical journals reported academic degrees and whether certain journal factors were associated with reporting academic degrees. Further research is needed to evaluate the impact of reporting academic degrees on readers’ perception of research.
- The following was rephrased in the Introduction section: “To our knowledge, *no evidence is available on how many journals choose to include academic degrees and whether this is more common in certain types of journals*.”
- We chose not to include a statement addressing that no evidence is available on the impact of reporting academic degrees following author names in medical journals in the Introduction section, since it may be out of scope with our aim of the current study. However, an appropriate statement has been provided in the Discussion section: “ *To our knowledge, no studies exist on the impact of reporting academic degrees in medical journals, why further research is needed to explore the implications of listing academic degrees on the readers’ attitude towards research quality.*”

 

**Comment 2:**

- “The description of the selection of articles in the set of 100 journals is vague. It is not clear what was the method for sampling "approximately ten articles ..." in the two study years. This needs to be clarified.”

 

**Response 2:**

- We agree that this definition needs to be clarified. We evaluated numerous original research articles in multiple journal issues to assess any discrepancy in reporting of academic degrees over the time period from July 2018 to August 2019. This would account to approximately 10 research articles. No discrepancy in reporting academic degrees during this time period within each journal was identified.
- Following was rephrased to clarify this issue in the Methods section: “ *Multiple articles published throughout July 2018 and August 2019 were assessed for each journal. No discrepancy of reporting academic degrees throughout the time period was identified.*”

# Peer review: Reviewer response for version 1

**DOI:** 10.5256/f1000research.23217.r56195

**Published:** 2019-11-08

**Authors:** Saif Aldeen AlRyalat

**Affiliations:** 1 Department of Ophthalmology, University of Jordan Hospital, The University of Jordan, Amman, Jordan

Stankovic et al assessed the factors associated with mentioning academic degrees on the journal’s title page. The current study is interesting and sheds a light on an important topic, although the authors chose a restricted sample to analyze (i.e. high impact medical journals). I believe that the current report can be improved by clarifying some points within its methods section, as follows.

 In the methods section, the authors did not define “academic degrees”. In the data associated with this study, they included columns on “Academic degrees”, “job titles”, and “fellowship designations”. In medicine, reporting of fellowships might be more important than the academic degree, especially if the researcher is a clinician working in a non-academic institution. The authors should clearly define what they mean by “academic degree” that they used in their analysis. 

 In the methods section, the authors categorized journals according to specialty as general or specific. The authors should detail the basis of this classification. Moreover, the authors categorized open access status into yes, no, or partly, where “partly” is not a common word used to describe the open access status (examples of common, well-defined words include green open access, hybrid access and so on). By going back to the data, I observed several non-open access journals categorized as “partly”, so I would suggest adopting strict criteria for categorization, such as the one suggested by Scopus:

 “Open Access Journals are indicated as Open Access if the journal is listed in the Directory of Open Access Journals (DOAJ) and/or the Directory of Open Access Scholarly Resources (ROAD)”.

 In the discussion section, the authors should include studies that discussed the impact of an academic degree in publishing, which would complement the points raised by the authors in the present report.

I confirm that I have read this submission and believe that I have an appropriate level of expertise to confirm that it is of an acceptable scientific standard, however I have significant reservations, as outlined above.

# Response

**Published:** 2019-12-06

**Authors:** Lars Andersen

**Affiliations:** Aarhus University Hospital, Denmark

**Comment 1: **

- **“**In the methods section, the authors did not define “academic degrees”. In the data associated with this study, they included columns on “Academic degrees”, “job titles”, and “fellowship designations”. In medicine, reporting of fellowships might be more important than the academic degree, especially if the researcher is a clinician working in a non-academic institution. The authors should clearly define what they mean by “academic degree” that they used in their analysis.”

 

**Response 1: **

- Thank you for this comment, which we agree will add clarification on the definition of “academic degrees”. Since “fellowship designations” were reported in six medical journals only - which all included academic degrees – we decided not to include a separate analysis for “fellowship designations”. Job titles were present in one medical journal, and this was available online only, why we chose to exclude this category from the final analysis.
- Thus, we included following clarification on the definition of academic degrees in the methods section of the manuscript: *“The presence of academic degrees was defined as the inclusion of academic degree abbreviations following author names (e.g. MD, PhD, RN, MPH etc.).”*

 

**Comment 2:**

- “In the methods section, the authors categorized journals according to specialty as general or specific. The authors should detail the basis of this classification.

 

**Response 2: **

- Agree. The medical journals were categorized as “general”, “specific” or “surgical” based on the specialty heterogeneity reported in the journal. Journals with high heterogeneity of reported specialties were categorized as “general”. In the statistical analysis, “surgical” was grouped with “specific”, since only two journals were categorized as “surgical”.
- However, to avoid equivocality, we have redefined the basis of our specialty classification for the journals. The journals are now classified according to subject area designation in Scopus. Consequently, the results have been adjusted according to this classification.
- Thus, we included following phrase in the methods section: “ *Journals were categorized as “general” or “specific” based on subject area designation in Scopus ^2^, i.e. whether content was primarily related to one specialty (e.g. cardiology) or multiple specialties.”*

 

**Comment 3**

- “Moreover, the authors categorized open access status into yes, no, or partly, where “partly” is not a common word used to describe the open access status (examples of common, well-defined words include green open access, hybrid access and so on). By going back to the data, I observed several non-open access journals categorized as “partly”, so I would suggest adopting strict criteria for categorization, such as the one suggested by Scopus: “Open Access Journals are indicated as Open Access if the journal is listed in the Directory of Open Access Journals (DOAJ) and/or the Directory of Open Access Scholarly Resources (ROAD)”.”

 

**Response 3:**

- Thank you for this insightful suggestion. We agree with the need to adopt strict criteria for categorization (such as Scopus). We have adopted Scopus’ definition of open access. The journals within the dataset have been reassessed according to Scopus’ definition of open access, and the current dataset, results and manuscript reflect the changes accordingly.
- Consequently, we have included following phrase in the methods section: *“Open access was identified based on open access designation in Scopus ^2^. Scopus registers open access as follows: “In Scopus, journals are registered as being OA journals only if they are registered as Gold OA or Subsidized OA at one or both of the following sources: Directory of Open Access Journals (DOAJ) and the Directory of Open Access Scholarly Resources (ROAD)” ^3^.”*

 

**Comment 4:**

- “In the discussion section, the authors should include studies that discussed the impact of an academic degree in publishing, which would complement the points raised by the authors in the present report.”

 

**Response 4:**

- Thank you for this comment. We agree that it would be valuable to include studies discussing the impact of an academic degree in publishing. However, to our knowledge, no studies have evaluated the impact of reporting academic degrees in journals, why it was not possible to include this in the discussion section.
- A statement has been provided in the Discussion section to address the lack of studies examining the impact of reporting academic degrees in publishing: “To our knowledge, *no studies exist on the impact of reporting academic degrees in medical journals, why further research is needed to explore the implications of listing academic degrees on the readers’ attitude towards research quality.”*
